# Council of the University of Buffalo

**Published:** 1882-03

**Authors:** 


### Council of the University of Buffalo.

At a meeting of the Council, held February 21st, the Hon. Orasmus H. Marshall was elected Chancellor, and Prof. Thomas F. Rochester, M. D., Vice-Chancellor. Hon. Sherman S. Rogers was elected a member of the Council to fill the vacancy occasioned by the death of Dr. James P. White. Dr. Mathew D. Mann was elected Professor of Obstetrics and Diseases of Women. The following memorial was adopted:

*Resolved*, That by the death of Dr. James P. White, one of the founders of our University, and a member of the Board, this institution has lost one of its ablest councilors and most zealous supporters.

We are largely indebted to his persevering efforts for the establishment of our Medical Department, and its growth to its present flourishing condition has been greatly promoted through his instrumentality.

*Resolved*, That we recognize with just pride the eminent professional abilities of the deceased, by which he acquired a national reputation, and we sympathize with his family, with his professional brethren, and with his survivors in the Medical Department in their common loss.

*Resolved*, That we deem it due to the memory of our associate, at the close of his honorable career, to bear testimony to his private worth and unsullied reputation. His marked decision of character and tenacity of purpose were strongly impressed on his public duties, and beneficially felt in the various benevolent and Christian enterprises in which he engaged.

*Resolved*, That these resolutions be transmitted to his family and published in the daily city papers.

